# Clinicopathological analysis of hepatic immune-related adverse events in comparison with autoimmune hepatitis and graft-versus host disease

**DOI:** 10.1038/s41598-021-88824-1

**Published:** 2021-04-29

**Authors:** Satoru Hagiwara, Tomohiro Watanabe, Masatoshi Kudo, Kosuke Minaga, Yoriaki Komeda, Ken Kamata, Masatomo Kimura, Hidetoshi Hayashi, Kazuhiko Nakagawa, Kazuomi Ueshima, Yasunori Minami, Tomoko Aoki, Masahiro Takita, Masahiro Morita, Hirokazu Cishina, Hiroshi Ida, Ah-Mee Park, Naoshi Nishida

**Affiliations:** 1 Department of Gastroenterology and Hepatology, Kindai University Faculty of Medicine, 377-2 Ohno-Higashi, Osaka-Sayama, Osaka 589-8511 Japan; 2 Department of Pathology, Kindai University Faculty of Medicine, Osaka, Japan; 3 Department of Medical Oncology, Kindai University Faculty of Medicine, Osaka, Japan; 4 Department of Microbiology, Kindai University Faculty of Medicine, Osaka, Japan

**Keywords:** Immunology, Gastroenterology

## Abstract

Immune checkpoint inhibitors (ICIs) targeting programmed cell death 1 (PD-1) and cytotoxic T-lymphocyte antigen-4 (CTLA-4) are widely used to treat advanced metastatic cancers. Neutralisation of PD-1 or CTLA-4 by ICIs results in immune-related adverse events (irAEs). The clinicopathological features of twelve patients with hepatic irAEs were evaluated and compared to those of ten patients with autoimmune hepatitis (AIH) or graft-versus-host disease (GVHD). No significant difference was seen in serum levels of transaminases, whereas serum levels of IgG and anti-nuclear antibody were higher in patients with AIH than in those with GVHD or hepatic irAEs. Inflammation was limited to the liver lobes in patients with GVHD or hepatic irAEs, whereas patients with AIH exhibited both portal and lobular inflammation. Immunohistochemical analyses revealed a predominant infiltration of CD8^+^ T cells and defective accumulation of regulatory T cells (Tregs) expressing forkhead box p3 (FOXP3) in the lobular areas of patients with hepatic irAEs and GVHD. In contrast, periportal lesions of patients with AIH were characterised by an infiltration of CD4^+^ T cells, CD8^+^ T cells, CD20^+^ B cells, and FOXP3^+^ Tregs. Overall, the activation of CD8^+^ T cells in the absence of activation of Tregs potentially underlies the immunopathogenesis of hepatic irAEs.

## Introduction

Remarkable progress has been made in chemotherapy against advanced metastatic cancers through the introduction of immune checkpoint inhibitors (ICIs)^1,2^. ICIs have been widely used for the treatment of advanced cancers, including melanoma, non-small-cell lung cancer, head and neck squamous cancers, urothelial carcinoma, and gastric adenocarcinoma^3–6^. Nivolumab and ipilimumab are representative ICIs targeting programmed cell death 1 (PD-1) and cytotoxic T-lymphocyte antigen-4 (CTLA-4), respectively. These ICIs bind to cell-surface PD-1 or CTLA-4 receptors expressed in T cells and restore anti-cancer immunity by promoting tumour antigen (Ag)-specific T cell responses^1,2^. Thus, it is clear that malignant tumours co-opt PD-1 and/or CTLA-4-mediated immune checkpoint pathways to avoid immune surveillance against tumours.

PD-1 and CTLA-4, both of which are expressed in activated T cells, negatively regulate immune responses via interaction with Ag-presenting cells expressing programmed death ligand 1 (PD-L1) and CD80/CD86, respectively. The molecular interactions between PD-1 and PD-L1 or CTLA-4 and CD80/CD86 effectively induces immune tolerance against self and non-self Ags^1,2^. Given the negative regulatory functions of PD-1 and CTLA-4, it is not surprising that the blockade of signalling pathways mediated by PD-1 and CTLA-4 may cause systemic autoimmune disorders through excessive immune responses to self and non-self Ags^7–9^. Such systemic autoimmune disorders caused by ICIs are called immune-related adverse events (irAEs)^7–9^. irAEs target a broad range of organs including the skin, endocrine system, lung, liver, kidney, gastrointestinal tract, and pancreas^7–9^.

Regulatory T cells (Tregs) are specialised CD4^+^ T cells characterised by the expression of the transcription factor forkhead box P3 (FOXP3)^10,11^. Tregs play essential roles in the maintenance of immune tolerance against self Ags, and impaired Treg function is responsible for the development of certain kinds of autoimmune diseases^10,11^. This is supported by the fact that impaired Treg function caused by loss of function mutations in *FOXP3* results in multi-organ autoimmune disease called immune dysregulation, polyendocrinopathy, enteropathy, X-linked syndrome (IPEX)^12^. IPEX causes excessive autoimmune responses in certain tissues, including the skin, endocrine system, pancreas, liver, kidney, and gastrointestinal tract^13^. Although the distribution of affected organs manifesting excessive immune reactions is shared by IPEX and irAEs, it remains largely unknown whether the accumulation of Tregs in the injured organs underlies the immunopathogenesis of irAEs, as in the case of IPEX. In this study, we investigated whether impaired accumulation of Tregs is involved in the development of hepatic irAEs. We evaluated the types of T cells, Tregs, and B cells accumulated in the hepatic lesions of patients with irAEs, and compared these findings to patients with autoimmune hepatitis (AIH) and graft-versus-host disease (GVHD).

## Methods

### irAEs patients

**Figure 1:**
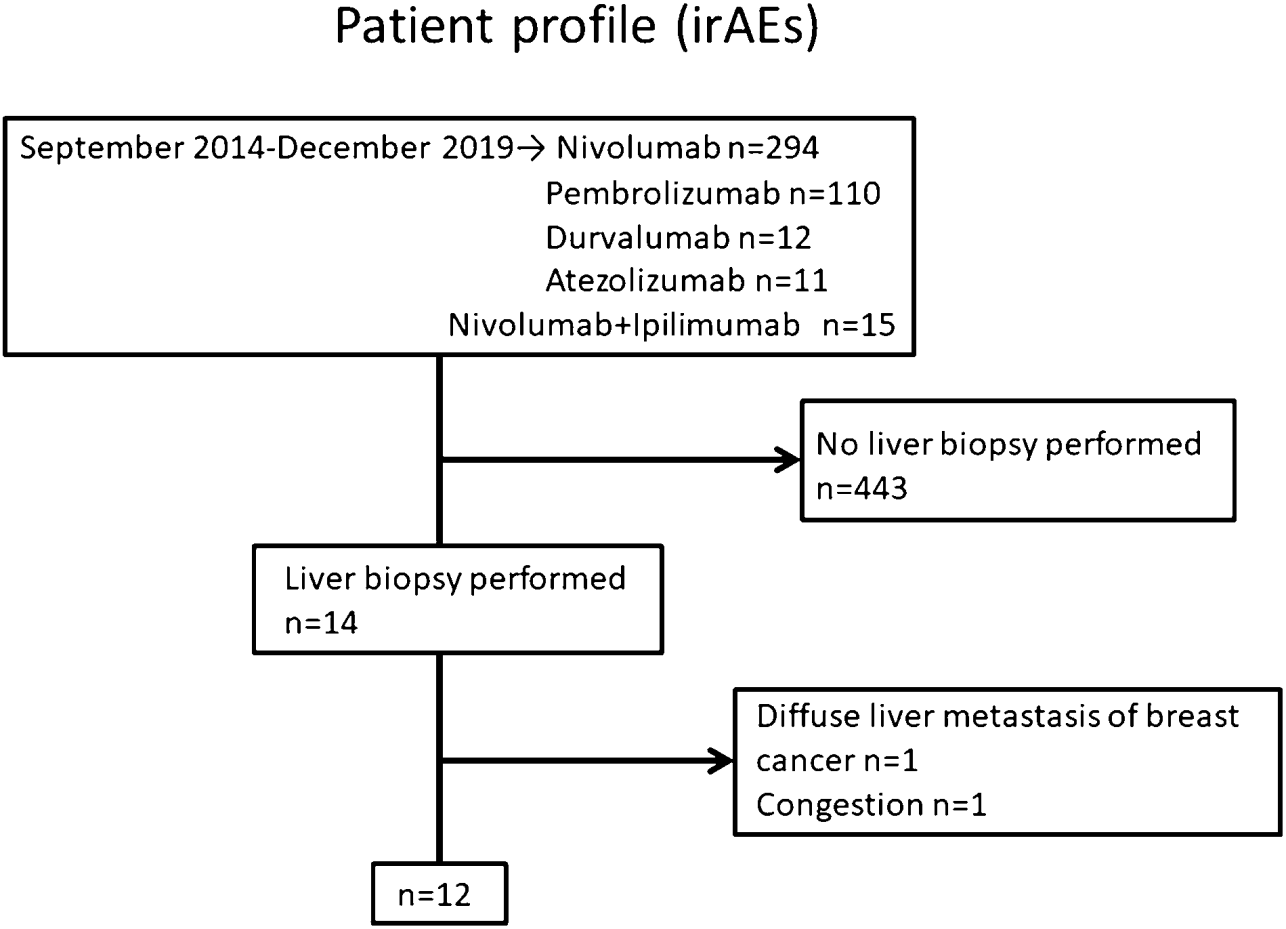
Selection of patients with hepatic irAEs. A total of 457 patients were treated with ICIs for advanced cancer at Kindai University Hospital from September 2014 to December 2019. Among these 457 patients, 14 were suspected of having hepatic irAEs based on the clinical course and elevation of serum transaminases, and subsequently underwent a liver biopsy. Twelve patients were diagnosed with hepatic irAEs.

Selection of irAEs patients was depicted in Fig. 1. A total of 457 patients were treated with ICIs for advanced cancer at Kindai University Hospital from September 2014 to December 2019. Of these 457 cases, 14 cases were suspected of having hepatic irAEs based on the clinical course and showed elevated serum transaminase or biliary enzyme of Grade3 or higher. Liver biopsy was performed for diagnostic purposes. Twelve patients were diagnosed with hepatic irAEs based on the pathological findings and serological screening for hepatitis A virus (HAV), HBV, HCV, cytomegalovirus, and Epstein-Barr virus. One case with diffuse metastasis of breast cancer and another case with congestive liver were excluded. Twelve patients with hepatic irAEs were included in this study.

### AIH patients and hepatic GVHD patients

Ten patients with definite AIH who met the diagnostic criteria of the 1999 revised edition of the international diagnostic criteria for AIH were included in this study^14^. Ten patients with pathologically-proven hepatic GVHD were also included. The diagnosis of hepatic GVHD was performed as previously described^15^.

### Ethics

The study protocol conformed to the ethical guidelines of the 1975 Declaration of Helsinki and was approved by the Institutional Review Board of Kindai University Faculty of Medicine (Approval Number 30-208). Written informed consent was obtained from all patients recruited in the study.

### Pathological analysis

Liver biopsy samples were subjected to hematoxylin and eosin (H&E) staining. Scoring of the portal, periportal, and lobular inflammation was performed as described previously^16^. Briefly, the pathological analyses were conducted using the METAVIR classification composed of inflammation within the bile duct, eosinophil infiltration, and the number of granulomas were evaluated as described previously^16^.

### Immunohistochemistry

Immunohistochemical analyses were performed using mouse or rabbit anti-human CD3 Ab (Roche Diagnostics; Tokyo, Japan), CD4 Ab (Roche Diagnostics; Tokyo, Japan), CD8 Ab (Nichirei Bioscience; Tokyo, Japan), CD20 Ab (Roche Diagnostics; Tokyo, Japan), CD56 Ab (Cell Signaling Technology, Danvers, MA), CD138 Ab (Agilent Technologies, Santa Clara, CA) and FOXP3 Ab (Abcam; Cambridge, United Kingdom). CD3^+^ T cells, CD4^+^ T cells, CD8^+^ T cells, CD20^+^ B cells, CD56^+^ NK cells, and FOXP3^+^ Tregs were visualized using DAKO-EnVision + systems (DAKO Japan, Tokyo, Japan) as previously described^17,18^. At least two immunohistochemical images were taken for each slide and the number of immune cells positive for each marker were counted in the high-powered fields.

### Immunofluorescence analyses

Dual immunofluorescence analyses were performed as described previously^17,18^. Each section was incubated with mouse or rabbit-CD3 Ab, CD4 Ab, CD56 Ab, or FOXP3 Ab followed by Alexa 488 or Alexa 546-conjugated rabbit or mouse IgG (Invitrogen, Carlsbad, CA). Nuclei were counter-stained with DAPI. At least two immunohistochemical images were taken for each slide and the number of immune cells positive for each marker were counted in the high-powered fields. Five liver biopsy samples obtained from AIH, GVHD, and hepatic irAEs were subjected to dual immunofluorescence analyses.

### Statistical analysis

Baseline characteristics are expressed as mean ± standard deviation for continuous variables. Evaluation of the differences between two groups for baseline characteristics was performed using the Students t-test. Similarly, the pathological score was also examined by Students t-test for comparison between the two groups. The pathological number of accumulated immune cells were examined by Mann–Whitney U-test for comparison between the two groups. All of the statistical analyses were performed using SPSS software (version 11.5; SPSS Inc., Chicago, IL, USA).

## Results

### Clinical characteristics of hepatic irAEs, AIH, and GVHD

The clinical characteristics of the twelve patients with hepatic irAEs enrolled in this study are shown in Table 1. ICIs were administered to nine patients with lung cancer, two with breast cancer, and one with malignant melanoma. Nivolumab (anti-PD-1 Ab) and pembrolizumab (anti-PD-1 Ab) were used in eight and three patients, respectively. One patient with malignant melanoma was treated with nivolumab and ipilimumab (anti-CTLA-4 Ab). Three of the 12 cases manifested the other organ involvement. With respect to the treatment, five patients were initially treated with prednisolone (PSL) ranging from 10 to 40 mg, whereas three patients were initially treated with mini-pulse with methyl PSL (500 mg) for three days followed by azathioprine or mycophenolic acid. One patient was treated with ursodeoxycholic acid, and three patients were observed without any treatment.

**Table 1:** Clinical patient background in hepatic irAEs patients.

No	Sex	Age	Type of cancer	ICI	Other organ involvement	Therapy for irAEs hepatitis	Immunosuppressive therapy at the time of liver biopsy
1	M	50	Lung	Anti-PD-1	Adrenal insufficiency/hypothyroidism/skin rash/interstitial pneumonia	mPSL 500 mg MMF 2000 mg	PSL 30 mg
2	F	83	Lung	Anti-PD-1	None	PSL 20 mg	None
3	F	89	Lung	Anti-PD-1	None	Follow-up	None
4	F	65	Lung	Anti-PD-1	Enteritis/pituitary dysfunction/rash	PSL10 mg	None
5	M	77	Lung	Anti-PD-L1	Acute renal failure/ acute pancreatitis	mPSL500 mg, AZA 50 mg HDF + PE	None
6	M	52	Lung	Anti-PD-1	None	mPSL 500 mg	None
7	M	80	Lung	Anti-PD-L1	None	PSL 40 mg	None
8	F	61	Breast	Anti-PD-1	None	Follow-up	None
9	F	68	Malignant Melanoma	Anti-PD1/ Anti-CTLA4	None	PSL 30 mg	None
10	M	68	Lung	Anti-PD-L1	None	Follow-up	None
11	M	75	Lung	Anti-PD-1	None	UDCA 600 mg	None
12	F	43	Breast	Anti-PD-1	None	PSL 30 mg	None

Anti-PD-1, anti-programmed cell death 1; Anti-CTLA4, anti-cytotoxic T-lymphocyte associated protein 4; PSL, prednisolone; MMF, mycophenolic acid; AZA, azathioprine; HDF, hemodiafiltration; PE, plasma exchange; UDCA, ursodeoxycholic acid.

The blood biochemical test results are shown in Table 2. No significant difference was seen in serum levels of aspartate aminotransferase (AST), alanine aminotransferase (ALT), and gamma-glutamyltransferase (γGTP) between hepatic irAEs, AIH, and GVHD patients, whereas these levels showed a higher trend in patients with AIH as compared with the other two disorders. Serum levels of IgG and anti-nuclear Ab titres were significantly higher in patients with AIH than in those with hepatic irAEs and GVHD. These results suggest that AIH, but not hepatic irAEs or GVHD, is characterised by enhanced Ab production, including total IgG and ANA.

**Table 2:** Comparison of background and liver damage data in irAEs, AIH, and GVHD patients.

	irAEs (n = 12)	AIH (n = 10)	GVHD (n = 10)
Age	#68 ± 14	#65 ± 11	*45 ± 13
Sex M/F	6/6	2/8	4/6
IgG	*1134 ± 278	#2634 ± 888	*1226 ± 719 (N.E.5)
ANA < 40/40/80/ ≥ 80	*12/0/0/0	#0/1/4/5	*4/0/1/0 (N.E.5)
AST	409 ± 584	613 ± 451	309 ± 244
ALT	443 ± 392	694 ± 578	424 ± 243
ALP	1175 ± 1213	571 ± 233	630 ± 410
γGTP	856 ± 1343	193 ± 70	318 ± 282
T-bil	1.3 ± 0.9	3 ± 2.8	6.9 ± 10
CRP	*4.8 ± 5.1	0.3 ± 0.2	1.3 ± 2.6

ANA, antinuclear antibody; AST, aspartate aminotransferase; ALT, alanine aminotransferase; CRP, C-reactive protein; N.E. not evaluated. *p* < 0.05 was defined as statistically significant.

### Pathological findings in hepatic irAEs, AIH, and GVHD

**Figure 2:**
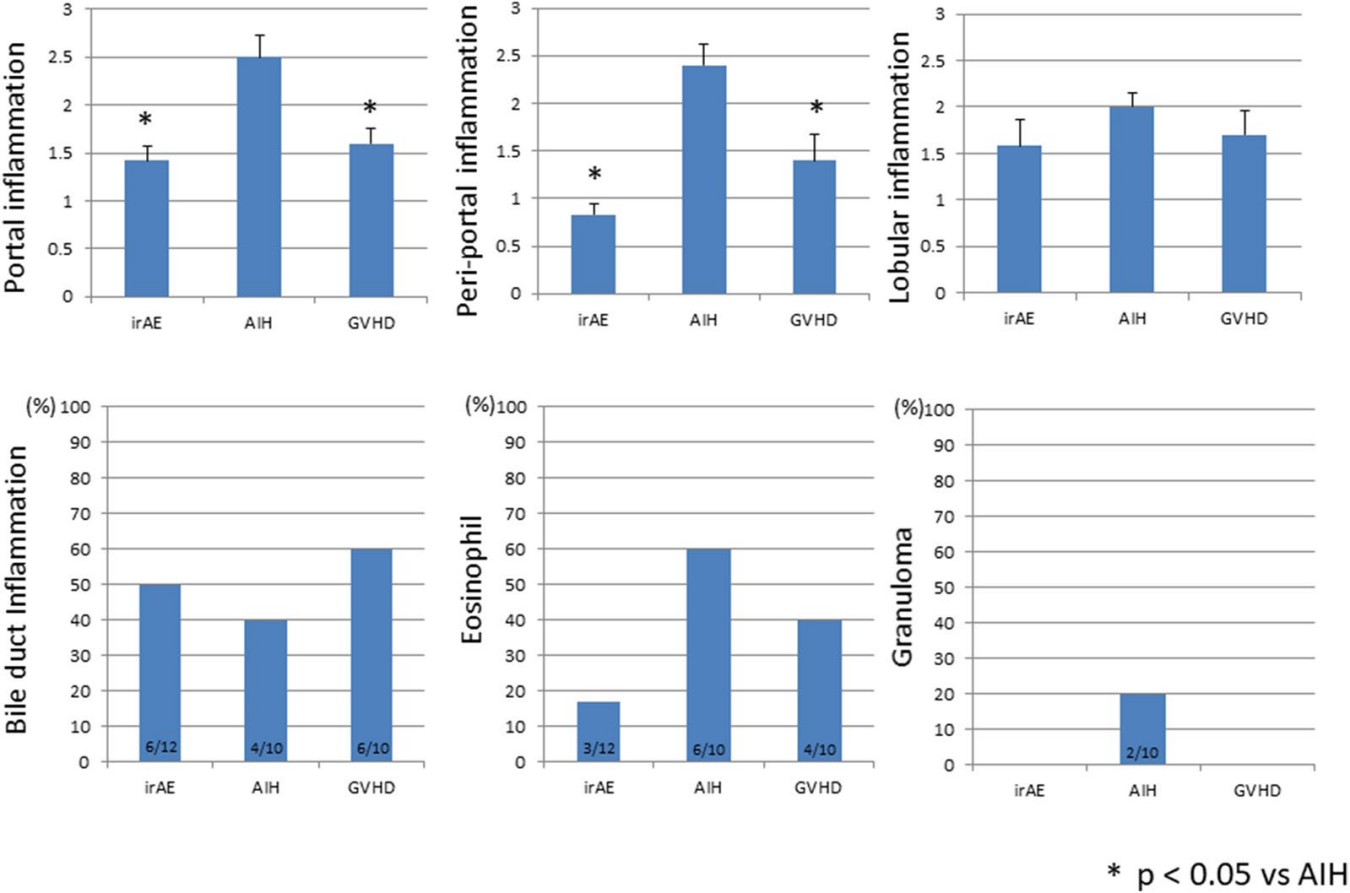
Pathological findings in hepatic irAEs, AIH, and GVHD. The degree of portal and periportal inflammation was evaluated in terms of portal inflammation, periportal inflammation, lobular inflammation, bile duct inflammation, eosinophil infiltration, and granuloma formation. The degrees of portal, peri-portal, and lobular inflammation were scored. The numbers of samples positive for bile duct inflammation, eosinophil infiltration, and granuloma formation were shown as percentages. *P < 0.05, as compared with AIH.

We next evaluated the severity of hepatic inflammation using a well-established scoring system in H&E staining. The degree of portal and periportal inflammation was greater in AIH than in hepatic irAEs or GVHD patients (Fig. 2). In contrast, the degree of lobular hepatitis was comparable between the three types of liver injury (Fig. 2). Moreover, the percentage of samples positive for bile duct inflammation were similar (Fig. 2), whereas those of samples positive for eosinophil infiltration were higher in AIH than in hepatic irAEs and GVHD patients. Thus, these pathological analyses using H&E staining revealed that liver injury associated with AIH, but not hepatic irAEs and GVHD, is characterised by portal and periportal inflammation. In addition, these pathological analyses also support the idea that hepatic irAEs shares pathological features with GVHD, in that lobular rather than portal inflammation is prominent. Lobular-predominant inflammation seen in patients with hepatic irAEs is fully consistent with previous reports^19,20^.

### Accumulation of adaptive immunity cells into the portal and periportal lesions in hepatic irAEs, AIH, and GVHD

**Figure 3:**
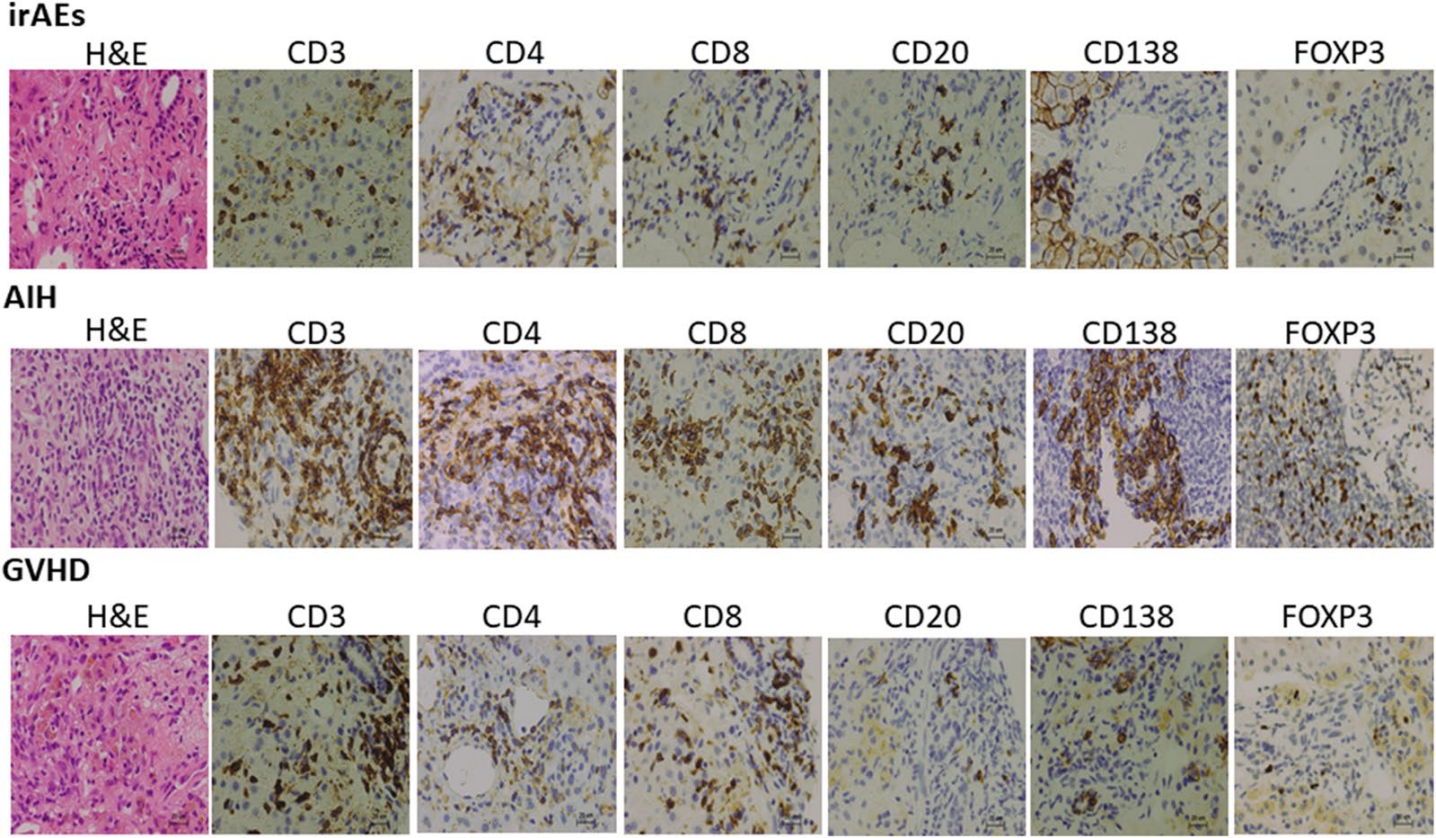
Accumulation of adaptive immune cells in the portal and periportal lesions of patients with hepatic irAEs, AIH, and GVHD. Liver biopsy samples were subjected to hematoxylin and eosin (H&E) staining and immuno-histochemical analysis to visualize CD3^+^ T cells, CD4^+^ T cells, CD8^+^ T cells, CD20^+^ B cells, CD138^+^ plasma cells and FOXP3^+^ Tregs. Infiltration of T cells, Tregs, and B cells was greater in the portal and periportal lesions of patients with AIH than in those of patients with hepatic irAEs and GVHD. Magnification × 400.

**Figure 4:**
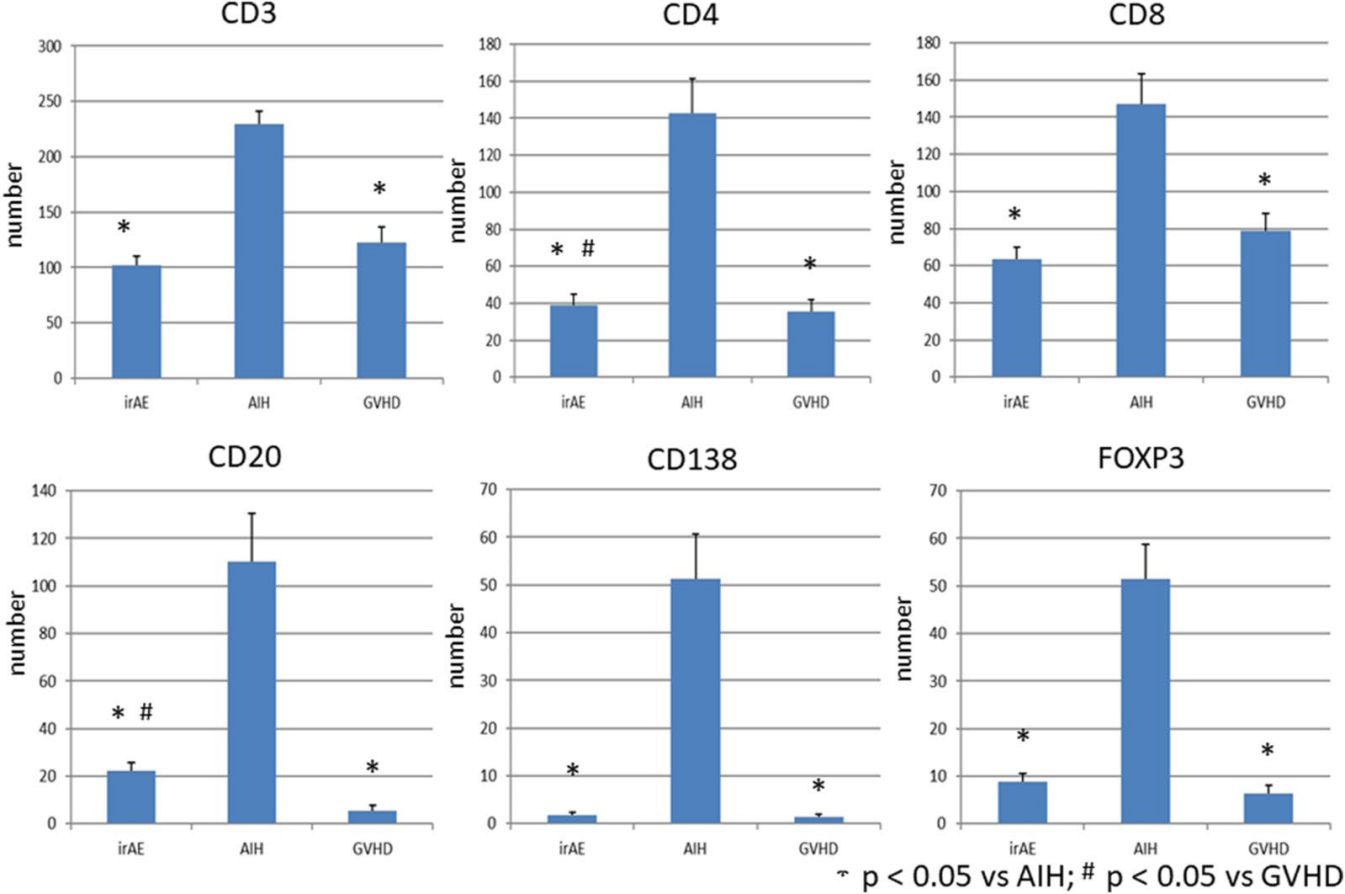
Semi-quantitative evaluation of immune cells that accumulated in the portal and periportal areas. The numbers of cells expressing CD3, CD4, CD8, CD20, CD138 and FOXP3 were counted in the portal and periportal areas of patients with hepatic irAEs, AIH, and GVHD in high powered fields. The results were shown as mean ± standard error. *P < 0.05, as compared with AIH. ^#^*P* < 0.05, as compared with GVHD.

Given that hepatic irAEs share pathological features with GVHD, but not AIH, we next evaluated the type of immune cells that accumulated in the inflammatory lesions. We visualized infiltration of CD3^+^ T cells, CD4^+^ T cells, CD8^+^ T cells, CD20^+^ B cells, CD138^+^ plasma cells and FOXP3^+^ Tregs. Massive accumulation of CD3^+^ T cells, CD4^+^ T cells, CD8^+^ T cells, CD20^+^ B cells, and CD138^+^ plasma cells was observed in the livers of patients with AIH. Abundant infiltration of T cells and B cells into the portal and periportal lesions was consistent with the enhancement of serum Ab responses in patients with AIH. It should be noted, however, that accumulation of Tregs expressing FOXP3 was also seen in the portal and periportal areas in AIH patients. In contrast, such accumulation of adaptive immune cells composed of CD3^+^ T cells, CD4^+^ T cells, CD8^+^ T cells, CD20^+^ B cells, CD138^+^ plasma cells and Tregs was significantly attenuated in the portal and periportal areas in hepatic irAEs or GVHD patients (Fig. 3). Semi-quantitative evaluation of immune cells that infiltrated into the portal and periportal areas also supported this finding (Fig. 4). Thus, these immunohistochemical analyses suggest that the accumulation of T cells, B cells, and Tregs is much lower in the portal and periportal areas of patients with hepatic irAEs and GVHD than in those of patients with AIH.

### Accumulation of adaptive immunity cells into the lobular lesions in hepatic irAEs, AIH, and GVHD

**Figure 5:**
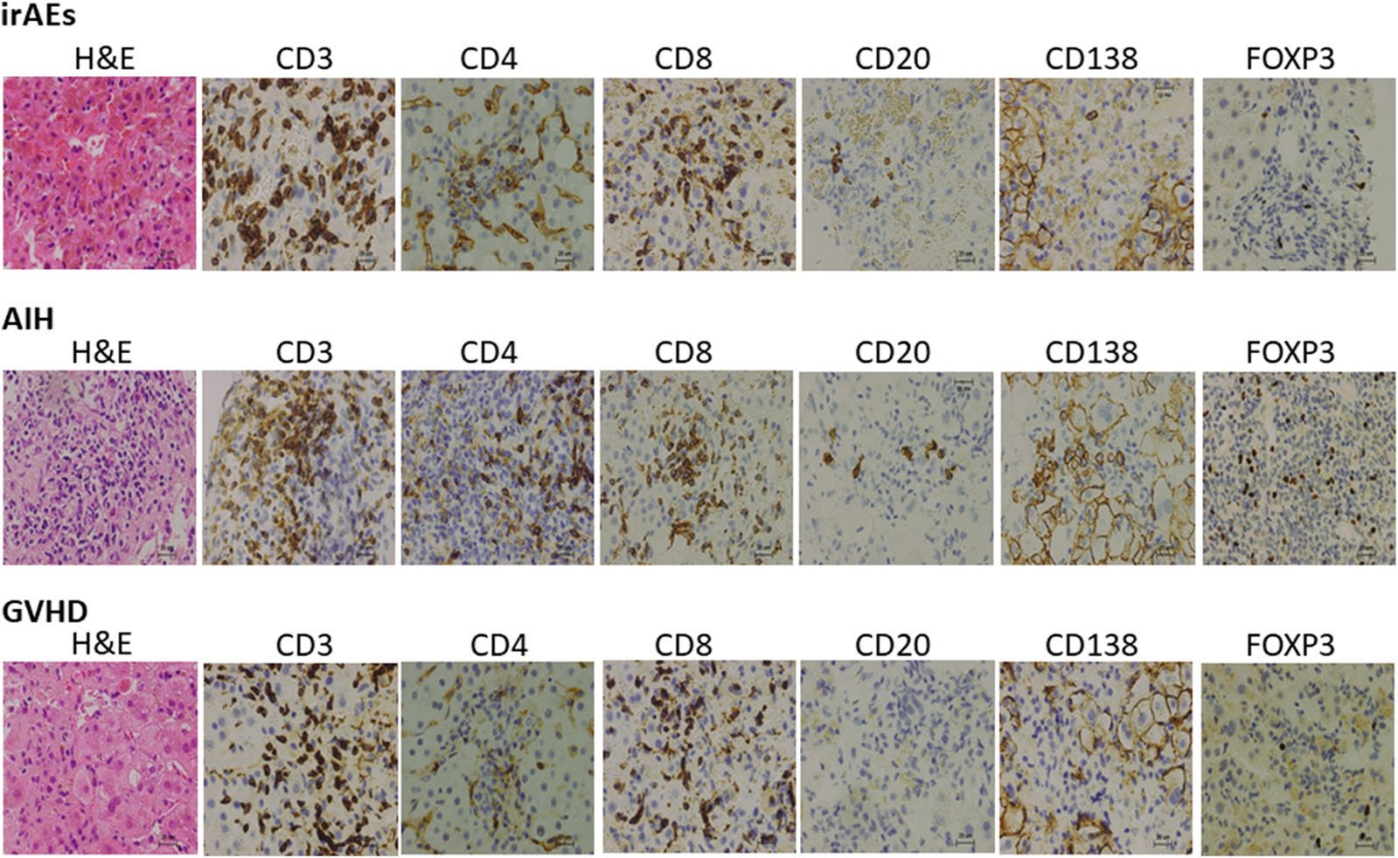
Accumulation of adaptive immune cells in the lobular lesions of patients with hepatic irAEs, AIH, and GVHD. Liver biopsy samples were subjected to hematoxylin and eosin (H&E) staining and immuno-histochemical analysis to visualize CD3^+^ T cells, CD4^+^ T cells, CD8^+^ T cells, CD20^+^ B cells, CD138^+^ plasma cells and FOXP3^+^ Tregs. Infiltration of CD3^+^ T cells and CD8^+^ T cells was comparable in the lobular lesions between hepatic irAEs, AIH, and GVHD whereas infiltration of Tregs expressing FOXP3 was greater in the lobular lesions of patients with AIH than in those of patients with hepatic irAEs and GVHD. Magnification × 400.

We then determined the type of adaptive immune cells that accumulated in the lobular areas of patients with hepatic irAEs, AIH, and GVHD. As shown in Fig. 5, the numbers of adaptive immune cells, including CD3^+^ T cells and CD8^+^ T cells were comparable in the lobular areas between patients with hepatic irAEs, GVHD, and AIH.

On the contrary, the numbers of cells expressing CD4 were markedly lower in patients with hepatic irAEs than in those with GVHD or AIH. It should be noted that the less infiltration of CD4^+^ T cells was seen in the lobular areas of patients with GVHD as compared with those with AIH. Thus, patients with hepatic irAEs are characterized by predominant inflammation of the lobular areas and by accumulation of CD8^+^ T cells, which findings were fully consistent with previous reports^19,20^.

**Figure 6:**
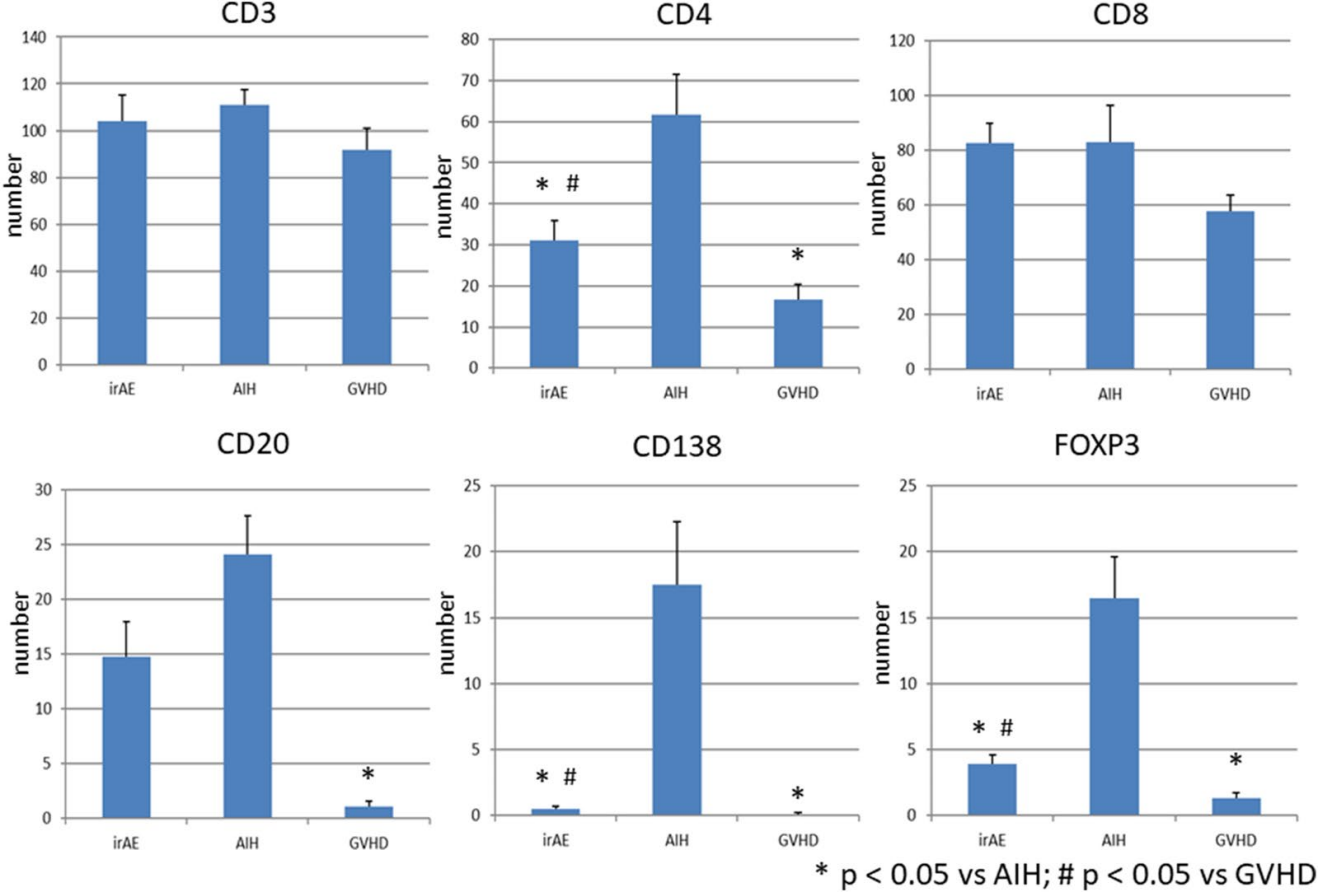
Semi-quantitative evaluation of immune cells that accumulated in the lobular areas. The numbers of cells expressing CD3, CD4, CD8, CD20, CD138 and FOXP3 were counted in the lobular areas of patients with hepatic irAEs, AIH, and GVHD in high powered fields. The results were shown as mean ± standard error. *P < 0.05, as compared with AIH. ^#^*P* < 0.05, as compared with AIH.

Although a moderate degree of infiltration of FOXP3^+^ Tregs was seen in the lobular areas of AIH patients, infiltration of FOXP3^+^ Tregs was barely seen in the lobular areas of patients with hepatic irAEs and GVHD. Thus, the accumulation of Tregs was barely seen not only in the portal and periportal areas but also in the lobular areas in the liver of patients with hepatic irAEs and GVHD, despite the fact that the degrees of inflammation and the numbers of accumulation of T cells and B cells are comparable to those of AIH. Semi-quantitative evaluation of immune cells infiltrated in the lobular areas also supported this finding (Fig. 6). Thus, these extensive immunohistochemical analyses support the idea that hepatic irAEs and GVHD are characterised by an impaired accumulation of Tregs into the portal, periportal, and lobular lesions.

### Dual immunofluorescence studies in the liver biopsy specimens from patients with AIH, GVHD, and hepatic irAEs

**Figure 7:**
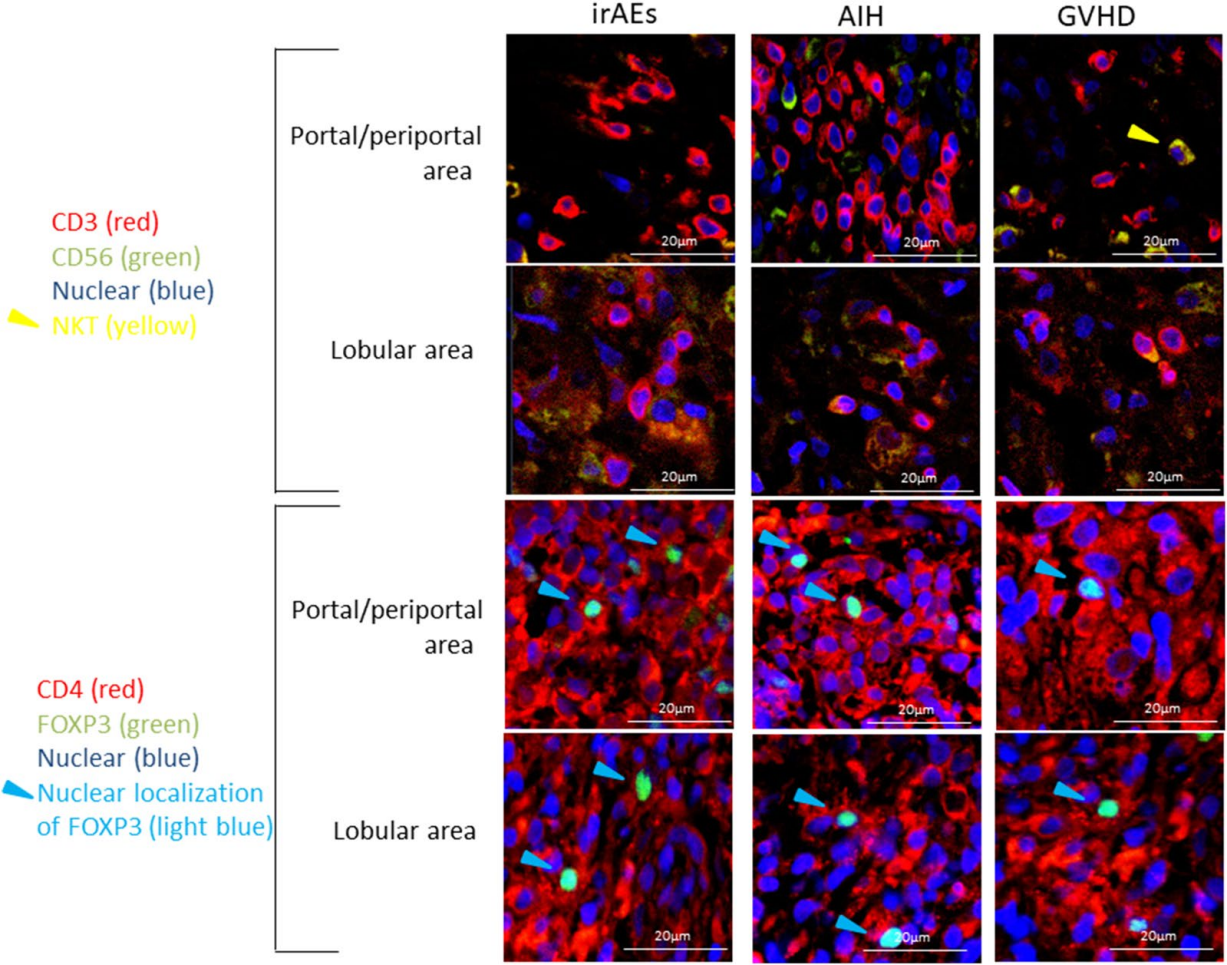
Dual immunofluorescence analyses of the liver biopsy samples. Liver biopsy samples from patients with AIH, GVHD, or hepatic irAEs (n = 5, each) were subjected to dual immunofluorescence analyses. Deparaffinized sections were incubated with mouse or rabbit CD3 Ab, CD4 Ab, CD56 Ab, or FOXP3 Ab followed by the incubation with Alexa 488 or 546-conjugated mouse or rabbit IgG. Nuclei were counterstained with DAPI. The results shown are one of the representative staining. CD3^+^CD56^+^ natural killer T (NKT) cells visualized yellow color were barely seen in the portal, periportal or lobular areas in patients with AIH, GVHD, or hepatic irAEs (1st row; portal and periportal area, 2nd row; lobular area). Almost all of the FOXP3^+^ cells were positive for CD4 staining (light blue color, 3rd row; portal and periportal area, 4th row; lobular area). Magnification × 630.

The liver is rich in natural killer (NK) T cells expressing both CD3 and CD56^21,22^. To exclude a possibility of NKT cell activation in the development of hepatic irAEs, liver biopsy samples were subjected to dual immunofluorescence analyses. As shown in Fig. 7, accumulation of CD3^+^CD56^+^ cells (NKT cells) visualized by yellow color was barely seen in the portal and /or lobular lesions in patients with AIH, GVHD, and hepatic irAEs.

We also performed dual immunofluorescence analyses to ascertain whether FOXP3^+^ cells are limited to CD4^+^ T cells. As shown in Fig. 7, FOXP3^+^ cells are exclusively positive for CD4 staining in the portal and /or lobular lesions in patients with AIH, GVHD, and hepatic irAEs. These immunofluorescence studies support our findings that hepatic lesions in patients with hepatic irAEs are characterized not only by abundant lobular infiltration of CD8^+^ T cells but also by impaired accumulation of CD4^+^FOXP3^+^ Tregs.

## Discussion

In this study, the types of T cells that accumulated in the lesions of patients with hepatic irAEs were characterised. Consistent with previous studies^20^, lobular rather than poral and/or periportal inflammation is predominant in patients exhibiting liver injury due to ICIs. Furthermore, immune cells that accumulated in the lobular lesions were mainly composed of CD8^+^ T cells, but not CD4^+^ T cells^19^.

Consistent with previous reports, predominant infiltration of CD8^+^ T cells into the lobular areas is one of the prominent features of hepatic irAEs. In addition to this finding, we found that lobular lesions of hepatic irAEs are characterised by the impaired accumulation of FOXP3^+^ Tregs. Thus, the present study provides evidence that both the accumulation of CD8^+^ T cells and impaired activation of Tregs are prominent features of hepatic irAEs exhibiting lobular hepatitis.

In comparison with AIH and GVHD, we found that the pathological findings of hepatic irAEs are similar to those of GVHD, but not AIH, in a few important points. First, the liver lobes in hepatic irAEs and GVHD patients are the main sites of damage. Second, CD8^+^ T cells rather than CD4^+^ T cells accumulate in the lesions of both diseases. Third, accumulation of FOXP3^+^ Tregs is impaired in both diseases. Thus, hepatic irAEs and GVHD are characterised by the accumulation of CD8^+^ T cells and impaired activation of Tregs. As shown in our dual immunofluorescence analyses, CD3^+^CD56^+^ NKT cells and Foxp3^+^ cells lacking CD4 expression were barely seen in the livers of patients with GVHD and hepatic irAEs. Therefore, conventional T cells rather than NKT cells accumulate into the hepatic lesions in patients with AIH, GVHD, and hepatic irAEs. Moreover, FOXP3 expression was limited to CD4^+^ T cells, suggesting that CD4^+^FOXP3^+^ Tregs are involved in the immuno-pathogenesis of AIH, GVHD, and hepatic irAEs.

It is well established that expansion of donor CD8^+^ T cells plays a critical role in the development of GVHD^23,24^. CD8^+^ T cells with strong cytotoxic T lymphocyte (CTL) activity mediate tissue injury through the expression of IFN-γ, granzyme, and perforin^23,24^. Importantly, loss of PD-1/PD-L1 interaction has been reported to be involved in the persistence of GVHD through expansion of donor CD8^+^ CTL T cells^25,26^. Thus, blockade of the PD-1/PD-L1 interaction leads to the expansion of CD8^+^ T cells reacting self and non-self Ags. Given the fact that all of the patients with hepatic irAEs were treated with nivolumab and pembrolizumab, it is likely that inhibition of intact PD-1-mediated signalling pathways sets the stage for the expansion of CD8^+^ T cells with CTL activity to promote liver injury. However, confirmation of this awaits phenotypical analyses of hepatic CD8^+^ T cells, that is, expression of IFN-γ, granzyme, and perforin, in patients with hepatic irAEs.

Tregs expressing FOXP3 play substantial roles in the maintenance of immune tolerance^27^. As shown in our immunohistochemical analysis, the development of lobular hepatitis seen in hepatic irAEs and GVHD patients is associated with the impaired accumulation of Tregs expressing FOXP3. In allogeneic bone marrow transplantation and GVHD, failure of development of Tregs is observed due to impaired thymopoiesis and propensity to death in the peripheral organs^28–30^. In line with this, the number of Tregs was much lower in GVHD than in AIH patients, and the number of Tregs in irAEs patients was equivalent to that of GVHD. Thus, impaired accumulation of Tregs in the liver lobes is a characteristic finding shared by hepatic irAEs and GVHD. Activation of PD-1 may be involved in such impaired activation of Tregs in GVHD and hepatic irAEs, as PD-1-deficient-Tregs display a more enhanced apoptosis than PD-1-intact-Tregs in the presence of low doses of interleukin-2, a crucial cytokine involved in Treg proliferation^28^. Moreover, neonatal thymectomy causes more severe liver injury in PD-1-deficient mice than in PD-1-intact mice through concurrent loss of Tregs expressing FOXP3^31^. In an orthotopic cancer model, transient FOXP3-expressing Treg depletion in combination with systemic administration of anti-PD-1 Ab successfully induced severe hepatic irAEs^32^. Taken together, these previous studies, together with our study, provide evidence that the neutralisation of PD-1 promotes liver injury through impaired activation of Tregs. Such a lack of Treg accumulation can partially explain the fact that the distribution of affected organs manifesting excessive immune reactions is shared by IPEX and irAEs, the former of which is caused by loss of function mutations in FOXP3.

In contrast to hepatic irAEs and GVHD, portal and periportal as well as lobular inflammation were seen in patients with AIH. Immune cells that accumulated in the portal/periportal lesions were composed of CD4^+^ T cells, CD8^+^ T cells, CD20^+^ B cells, CD138^+^ plasma cells and FOXP3^+^ Tregs. Thus, various adaptive immune cells infiltrate the portal and periportal lesions of patients with AIH, suggesting that maturation of immune networks in the portal tract is necessary for the development of AIH^33^. In addition, CD4^+^ T cells and CD20^+^ B cells accumulated into the lobular lesions of patients with AIH, but not hepatic irAEs or GVHD. Compatible to previous studies^34–37^, FOXP3^+^ Tregs migrate into the lesions of patients with AIH. Given that patients with AIH, but not hepatic irAEs or GVHD, are characterised by elevated serum concentrations of IgG and ANAs, the production of pathogenic autoAbs requires the formation of portal as well as lobular immune networks in AIH. Therefore, the degree of portal and/or lobular immune-network maturation might be associated with the development of AIH, GVHD, and hepatic irAEs.

In conclusion, hepatic irAEs are characterised by lobular inflammation mediated by CD8^+^ T cells and is accompanied by the impaired activation of Tregs expressing FOXP3. These pathological features are similar to those of GVHD, but not AIH. Further functional studies utilising CD8^+^ T cells and Tregs isolated from patients with hepatic irAEs are required to verify these findings.
